# PFOA and Cancer in a Highly Exposed Community: New Findings from the C8 Science Panel

**DOI:** 10.1289/ehp.121-A340

**Published:** 2013-12-01

**Authors:** Wendee Nicole

**Affiliations:** Wendee Nicole was awarded the inaugural Mongabay Prize for Environmental Reporting in 2013. She writes for Discover, Scientific American, National Wildlife, and other magazines.

Past laboratory research has associated perfluorooctanoic acid (PFOA) with liver, testicular, and pancreatic cancers in rodents.^1^ Human studies of PFOA have lacked statistical power, although one study did find a significant association between kidney cancer deaths and serum levels of PFOA in chemical plant workers.^2^ Now a major epidemiological study published in *EHP* reports an association between PFOA exposure and kidney and testicular cancers in individuals who lived near and worked at a plant that produced the chemical.^3^

PFOA is found in the blood of an estimated 98% of Americans.^4^ The chemical is used in the manufacture of items such as Teflon® nonstick coating, Gore-Tex® water-repellent gear, microwave popcorn bags, carpet, and fire-fighting foam. The eight main fluoropolymer manufacturers, including DuPont, participate in the U.S. Environmental Protection Agency’s 2010/2015 PFOA Stewardship Program, which seeks to eliminate PFOA and related chemicals from products and factory emissions by 2015.^5^

The present study arose from a 2001 class action lawsuit in which residents living near DuPont’s Washington Works plant on the Ohio–West Virginia border sued the company for contaminating groundwater with PFOA over several decades. The suit led to an unusual settlement agreement: A panel of three epidemiologists appointed by the Circuit Court of Wood County would study whether PFOA (also called C8^6^) caused various health outcomes. If the C8 Science Panel found any disease was “more probably than not” associated with PFOA exposure—a legal term rather than a scientific one—DuPont would pay for ongoing medical monitoring for those ailments.^7^

Such research isn’t usually part of this type of settlement, says panel member Kyle Steenland, an Emory University professor of epidemiology. Companies may settle a case, but no scientific evidence is gathered to determine whether the chemical actually caused the problem.

The C8 Science Panel studied 55 health outcomes and, between 2011 and 2012, delivered four reports to the court concluding that PFOA was probably linked to six outcomes: kidney cancer, testicular cancer, ulcerative colitis, thyroid disease, hypercholesterolemia, and pregnancy-induced hypertension.^8^ The current study came out of the panel’s work.

**Figure:**
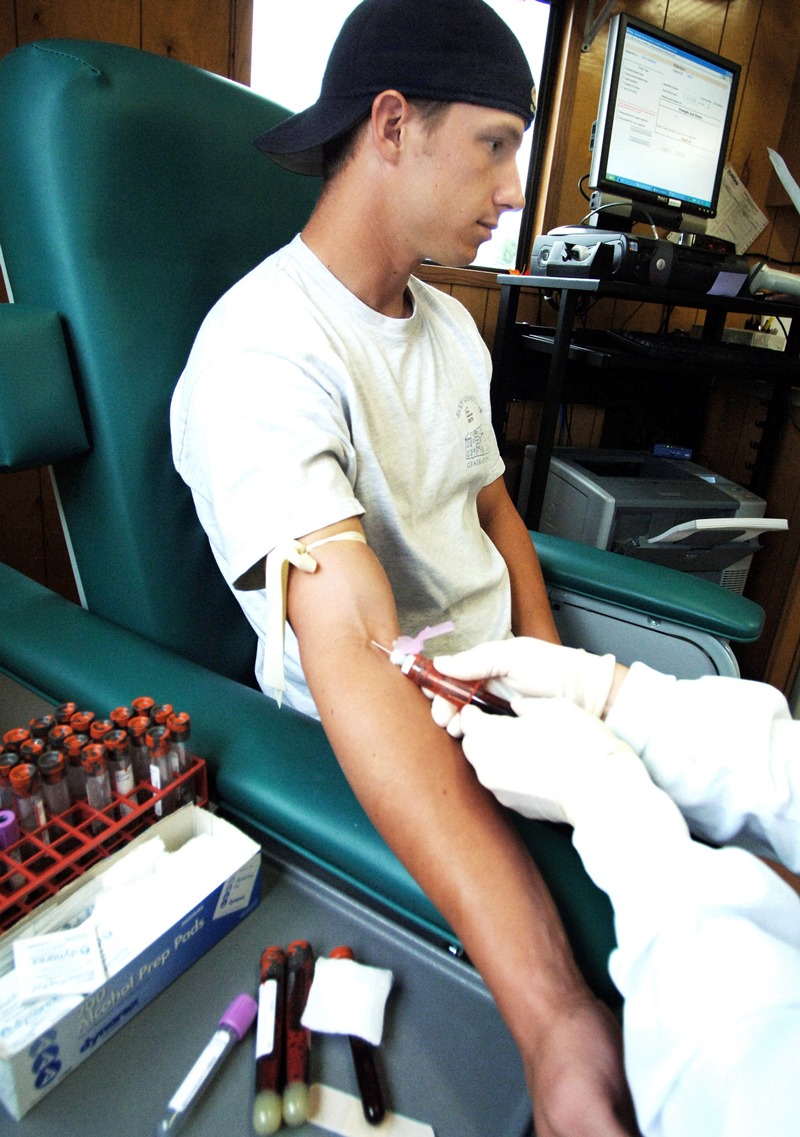
A participant in the C8 Health Project has blood drawn during baseline screening in 2005. © AP Photo/Jeff Gentner

Steenland and colleagues used health data collected from community residents in 2005 and 2006 and from a follow-up medical survey of these participants between 2008 and 2011. They also included data from 4,391 DuPont workers. For each worker and resident, the authors estimated lifetime cumulative PFOA serum levels based on factors including drinking water source, tap water consumption, and any employment at the DuPont plant.

Of 32,507 individuals in the current study, 2,507 had primary cancers of 21 different types that were validated using medical records and cancer registries. The incidence of both testicular and kidney cancers increased with higher estimated PFOA serum levels. Although the dose–response trend was not statistically significant for kidney cancer, the association is supported by two other studies by the C8 Science Panel: a geographical study of cancer in the mid-Ohio Valley^9^ and a mortality study of DuPont workers.^2^

The authors also found a positive association between estimated PFOA levels and thyroid cancer, but there was not a consistent dose–response trend, and the association was not statistically significant. Thyroid cancer was of interest because the C8 Science Panel had previously concluded that a “probable link” exists between PFOA and noncancerous thyroid diseases such as hypo- and hyperthyroidism, which affect metabolism.^10^ It was unclear whether these conditions were linked to thyroid cancer.

“This study shows how many of the end points affected by PFOA in rodents appear to be affected by exposures in human populations,” says Laura Vandenberg, an assistant professor of environmental health science from the University of Massachusetts Amherst, who was not involved in the study. “The strength of the study is that it largely corroborates what was already known from animal studies.”

“There are admittedly some limitations in study design, most notably that it is [largely] a survivor cohort, as the authors note,” says Emily Barrett, an assistant professor at the University of Rochester Medical Center, who also was not involved in the study. That is, community members who developed cancer had to survive until 2004–2005 to be eligible for the C8 cohort, although former workers could be counted as cases even if they died before the study.

Consequently, community members who died of fast-moving cancers were less likely to be included in the study, which could result in an underestimate of the association between PFOA and that cancer. On the other hand, associations could be overestimated since people with cancer who knew they had been highly exposed may have had a greater incentive to enroll in the study. The small numbers of cases for some individual cancers, including testicular cancer, also may have led to imprecise estimates of association.

Data from the nationally representative National Health and Nutrition Examination Survey 1999–2000 showed that participants averaged PFOA blood concentrations of 5.2 ng/mL,^4^ whereas the cohort in the six counties exposed to contaminated drinking water near the DuPont Washington Works plant had concentrations averaging 32.9 ng/mL.^11^ “I think that it will be important to next look at C8 exposure and cancer risk in the general population rather than high-exposure cohorts,” says Barrett. “Those analyses will be most relevant to weighing risks to the general public.”
